# Chemical Composition and Plant Growth of *Centaurea raphanina* subsp. *mixta* Plants Cultivated under Saline Conditions

**DOI:** 10.3390/molecules25092204

**Published:** 2020-05-08

**Authors:** Spyridon A. Petropoulos, Ângela Fernandes, Maria Ines Dias, Carla Pereira, Ricardo C. Calhelha, Antonios Chrysargyris, Nikolaos Tzortzakis, Marija Ivanov, Marina D. Sokovic, Lillian Barros, Isabel C. F. R. Ferreira

**Affiliations:** 1 Department of Agriculture Crop Production and Rural Environment, University of Thessaly, Fytokou Street, 38446 N. Ionia, Magnissia, Greece; 2 Instituto Politécnico de Bragança, Campus de Santa Apolónia, Centro de Investigação de Montanha (CIMO), 5300-253 Bragança, Portugal; afeitor@ipb.pt (Â.F.); maria.ines@ipb.pt (M.I.D.); carlap@ipb.pt (C.P.); calhelha@ipb.pt (R.C.C.); lillian@ipb.pt (L.B.); 3 Department of Agricultural Sciences, Biotechnology and Food Science, Cyprus University of Technology, 3603 Lemesos, Cyprus; a.chrysargyris@cut.ac.cy (A.C.); nikolaos.tzortzakis@cut.ac.cy (N.T.); 4 Institute for Biological Research “Siniša Stanković”-National Institute of Republic of Serbia, University of Belgrade, Bulevar despota Stefana 142, 11000 Belgrade, Serbia; marija.smiljkovic@ibiss.bg.ac.rs (M.I.); mris@ibiss.bg.ac.rs (M.D.S.)

**Keywords:** abiotic stress, antimicrobial properties, antioxidants, cytotoxicity, flavanones, oxalic acid, polyphenols, pinocembrin, saline conditions

## Abstract

The aim of this report was to study the effect of salinity (control: 2dS/m, S1: 4 dS/m and S2: 6 dS/m) and harvest time (first harvest on 9 May 2018 and second harvest on 19 April 2018) on the growth and the chemical composition of *Centaurea raphanina* subsp. *mixta* plants. The plants of the first harvest were used for the plant growth measurements (fresh weight and moisture content of leaves, rosette diameter, number and thickness of leaves), whereas those of the second harvest were not used for these measurements due to the flowering initiation, which made the leaves unmarketable due to their hard texture. The results of our study showed that *C. raphanina* subsp. *mixta* plants can be cultivated under mild salinity (S1 treatment) conditions without severe effects on plant growth and yield, since a more severe loss (27.5%) was observed for the S2 treatment. In addition, harvest time proved to be a cost-effective cultivation practice that allows to regulate the quality of the final product, either in edible form (first harvest) or for nutraceutical and pharmaceutical purposes as well as antimicrobial agents in food products. Therefore, the combination of these two agronomic factors showed interesting results in terms of the quality of the final product. In particular, high salinity (S2 treatment) improved the nutritional value by increasing the fat, proteins and carbohydrates contents in the first harvest, as well as the tocopherols and sugars contents (S1 and S2 treatments, respectively) in the second harvest. In addition, salinity and harvest time affected the oxalic acid content which was the lowest for the S2 treatment at the second harvest. Similarly, the richest fatty acid (α-linolenic acid) increased with increasing salinity at the first harvest. Salinity and harvest time also affected the antimicrobial properties, especially against *Staphylococcus aureus*, *Bacillus cereus* and *Trichoderma viride*, where the extracts from the S1 and S2 treatments showed high effectiveness. In contrast, the highest amounts of flavanones (pinocembrin derivatives) were detected in the control treatment (second harvest), which was also reflected to the highest antioxidant activity (TBARS) for the same treatment. In conclusion, *C. raphanina* subsp. *mixta* plants seem to be tolerant to medium salinity stress (S1 treatment) since plant growth was not severely impaired, while salinity and harvesting time affected the nutritional value (fat, proteins, and carbohydrates) and the chemical composition (tocopherols, sugars, oxalic acid, fatty acids), as well as the bioactive properties (cytotoxicity and antimicrobial properties) of the final product.

## 1. Introduction

The Mediterranean Basin is abundant in several wild and domesticated horticultural species, which show great adaptation abilities to arduous conditions, such as the high salinity environments of coastal and/or intensively cultivated regions [1,2]. Moreover, the broader Mediterranean region is characterized by small-scale farming which is the back-bone of the farming sector and of rural economies and is under increasing pressure due to land allocation for non-agricultural uses and to the ongoing climate change [2,3,4]. The ability of wild species to resist or tolerate stressful conditions suggests they could be used as alternative farming options in soils and climates where conventional species are difficult or impractical to be grown; for the same reason, these species can become an option for small-scale farming allowing the farmers to be competitive within the rapidly changing global market and market needs for novel foods [5,6,7,8,9,10]. Until recently, many reports have highlighted the importance of growing conditions and cultivation practices for producing wild edible greens, focusing on the domestication of these species, their incorporation in sustainable cultivation systems and the rational use of natural resources [2,11,12,13,14,15]. Moreover, several efforts have been made to emphasize the health benefits resulting from the inclusion of these species in modern diets, since they usually contain numerous bioactive secondary metabolites associated with positive health effects [16,17,18,19]. Furthermore, before safely suggesting such novelty species to the general public, special attention should be given to any potential toxic effects because many of these wild edible greens may contain anti-nutritional and toxic factors [7,20,21,22,23].

Among the various genera of the Asteraceae family, the *Centaurea* genus includes various species, with several of them being endemic to specific regions, while their uses also show great diversity, with several of them being edible or others being used for medicinal purposes in rural communities [24,25,26,27,28]. So far, there are several reports that refer to the chemical composition and bioactive properties of *Centaurea* species, most of which study wild plants collected from their natural environments [18,29,30,31,32]. The most interesting compounds reported in this genus are sesquiterpene lactones [33,34], while it also contains phenolic compounds [30,35], tocopherols [36] and fatty acids [37,38]. *Centaurea raphanina* subsp. *mixta* (DC.) Runemark (also known as agginaráki or alivárvaron [39]) is a perennial species of the Asteraceae family, which is native in the Mediterranean Basin and endemic to Greece and resembles the flowers of globe artichoke, both of which belong to the same tribe Cynarae [40,41,42]. It is believed that its name refers to Chiron, who according to Greek mythology was the leading centaur with exceptional knowledge in botany and the therapeutical properties of herbs. The edible tender leaves of *C. raphanina* subsp. *mixta* are a common ingredient of the Mediterranean diet and are highly appreciated for their distinct bitter flavour [39,43,44]. However, there is limited information regarding the nutritional value and the chemical composition of *C. raphanina* subsp. *mixta* [40,45], whereas no literature reports exist regarding the effects of cultivation practices on the plant growth and on the chemical composition and bioactive properties of the edible plant parts. According to Fernández-Marín et al. [46], the domestication of wild species may have a variable effect on specific compounds such as tocopherols and fatty acids, while recently Namzer et al. [47] reported significant variation in the chemical profile of wild and domesticated purslane plants, some with positive (e.g., higher vitamin K and E and lower content of oxalic acid in cultivated plants) and others with negative signs (e.g., lower content of total polyphenols in cultivated plants). Similarly, Disciglio et al. [11], who studied the chemical composition of wild and cultivated *Borago officinalis* L., *Cichorium intybus* L. and *Diplotaxis tenuifolia* L. plants, reported higher contents of total phenols and antioxidant activity for wild *C. intybus* and *B. officinalis* plants, whereas no significant differences were reported in the case of *D. tenuifolia*. Moreover, cultivation practices such as the fertilization regime, the salinity level, the harvesting stage and the growing medium could affect the chemical composition and the overall quality and the bioactive properties of the final product [2,8,14,15,48,49].

Within the concept of saline agriculture and the ongoing climate change, several species have been proposed during recent years as candidate cash crops including *Cichorium spinosum,* purslane, Salicornia, sea fennel and cardoon, among others. [50,51,52,53,54]. However, finding new crops that can be cultivated as alternative/complementary species is essential to cover the increasing consumers’ needs and the ever-widening market niches. For this purpose, the aim of the present study was to test the response of *C. raphanina* subsp. *mixta* plants to saline conditions and evaluate the potential of using the species as a new cash crop in saline environments. Furthermore, we studied the impact of salinity and harvest time on the chemical composition and the bioactive properties of the species, aiming to identify those cultivation practices that could increase its bioactivity and improve its overall quality through the regulation of the chemical profile of leaves.

## 2. Results and Discussion

### 2.1. Plant Growth

Parameters of the agronomic performance of *C. raphanina* subsp. *mixta* cultivated under saline conditions are presented in Table 1. Results from the second harvest are not presented due to early anthesis prior to harvesting, since leaves are not considered edible at this stage due to their hard texture. The fresh weight of the plants was significantly reduced with the increasing electric conductivity (EC) of the nutrient solution by 11.7% and 27.5% for the S1 and S2 treatments, respectively. Similar trends were recorded for the number of leaves, indicating that the fresh weight loss is partly attributed to the formation of less leaves rather than smaller leaves, although no significant differences were observed for these parameters, as well as for differences in the moisture content (see Table 2). This is further justified by the fact that the rosette diameter and leaf thickness were not affected by the salinity level, which could also be the result of heterogeneity in the morphology among the plants of wild ecotypes [55]. Despite the yield losses under saline conditions observed in our study, it has to be considered that the medium level tested (S1: 4.0 dS/m) which provoked a small yield loss is usually prohibitive for plant growth of many conventional leafy crops and results in severe losses of yield [56,57]. So far, there are reports regarding the response of other *Centaurea* genus members to salinity stress [58,59,60,61], including some halophytic species of the genus [62,63]. However, to the best of our knowledge this is the first report regarding the impact of salinity on plant growth of *C. raphanina* subsp. *mixta*. Therefore, the findings of the present study show promising potential for introducing the species as a new cash crop to mildly salt-affected soils and groundwater reservoirs, since the observed results do not indicate tolerance to high salinity levels, such as those tested in our study (S2: 6.0 dS/m). 

### 2.2. Chemical Composition

Regarding the combinatory effect of the tested factors, a significant interaction was observed for all the tested parameters. Therefore, in the next sections, only the two-way ANOVA results will be presented.

#### 2.2.1. Proximate Analysis and Energetic Value

The results of the proximate analysis and the energetic value of *C. raphanina* subsp. *mixta* leaves in relation to salinity level and harvesting time are presented in Table 2. The highest moisture content was observed in the control treatment of the second harvest (90.6 g/100 g fw), whereas decreasing trends in the moisture content were observed with increasing salinity levels. In contrast, most the tested parameters had the lowest values in the control treatment of the second harvest, which indicates a concentration effect, since the highest values were generally observed in the S2 treatment of the first harvest where the lowest moisture content was recorded. The presented results were within the same range of a recent report of our team where cultivated and wild *C. raphanina* subsp. *mixta* were compared [40], although the carbohydrates content and the energetic value of the present study were lower than the wild plants in the study of Petropoulos et al. (9.7 g/100 fw of carbohydrates and 54.1 kcal/100 g fw) [40]. Therefore, it seems that cultivation practices such as saline irrigation water and harvesting time may affect the nutritional value and macronutrients content of *C. raphanina* subsp. *mixta* mostly through the increase of dry matter content and the resulting concentration effect. Similar trends were reported for conventional crops such as lettuce, where harvesting time and salinity affected the nutritional value [64,65], or cauliflower and artichoke, where increasing salinity resulted in an increased dry matter content [66,67]. The present findings suggest consumers could consume lower amounts of fresh edible leaves from plants subjected to salinity stress, especially those harvested early in the season, in order to cover a percentage of the recommended daily allowance [7]. This is very important considering the use of wild edible greens as gourmet delicacies in various dishes. 

#### 2.2.2. Tocopherols Content

The tocopherols composition is presented in Table 3, where the α- and γ-tocopherols were the only detected isoforms of vitamin E, a finding that is in accordance with the recent report of Petropoulos et al. [40], who also detected the same compounds. However, the individual and total tocopherols content was higher in our study, especially under saline conditions where a two- to three-fold increase was observed compared with the cultivated plants in the study of Petropoulos et al. (0.185 mg/100 g, 0.067 mg/100 g and 0.26 mg/100 g fw of α-, γ- and total tocopherols, respectively) [40]. The most abundant tocopherol was α-tocopherol which was the richest in leaves of the second harvest, especially in the control and S1 treatments (0.68 mg/100 g and 0.69 mg/100 g fw, respectively), where the highest content of total tocopherols was also observed (0.73 mg/100 g and 0.74 mg/100 g fw, respectively). In contrast, the highest content of γ-tocopherol was observed in the first harvest and the S1 treatment. Moreover, the tocopherols content increased with increasing salinity only in the first harvest, whereas the opposite trend was observed in the second one. This finding could be due to the fact that saline conditions induced the tocopherols composition in the first harvest as a part of the antioxidant mechanism of the plant against salinity stress [68,69], as well as to increasing temperatures which have been associated with an increased α-tocopherol content [49]. Moreover, the fact that plants of the second harvest were in the reproductive stage also has to be considered, since the developmental stage is associated with the tocopherols content and composition in wild edible greens [14,49] and other crops [70]. Therefore, considering that leaves at this stage are usually not suitable for culinary uses, the increased content in tocopherols and α-tocopherol in particular shows great potential for alternative uses in nutraceuticals.

#### 2.2.3. Free Sugars Content

The sugars composition of *C. raphanina* subsp. *mixta* as affected by the harvesting time and the salinity level of the nutrient solution is presented in Table 4. Fructose, trehalose, glucose and sucrose were the only sugars detected in the tested samples in amounts that varied depending on the harvesting time and the salinity level. The highest content of total sugars (1.19 g/100 g fw) was recorded for the S2 treatment of the second harvest due to the high content of fructose and trehalose (0.50 g/100 g and 0.322 g/100 g fw, respectively). The sucrose and glucose contents were the highest in the S2 treatment of the first harvest (0.216 g/100 g fw) and the control treatment of the second harvest (0.285 g/100 g fw), respectively. The detected amounts of fructose, glucose and trehalose in the control treatment were in the same range as in the report of Petropoulos et al. for cultivated plants [40], whereas the sucrose content was higher in our study resulting in a higher total sugars content compared with the previous report. These differences could be ascribed to differences in the growing conditions, since in our study harvest took place in March (first harvest) and April (second harvest), whereas in the study of Petropoulos et al. [40], cultivated plants were harvested in October where different light quality and intensity conditions occurred [71]. Moreover, in our study, the plants were cultivated in an unheated plastic greenhouse compared with the field conditions implemented in the study of Petropoulos et al. [40]. The overall increase of free sugars under high salinity (S2) in the first and second harvests also indicates the significant role of such osmolytes as osmoprotective agents against stress conditions [5], as well as in the biosynthesis of secondary metabolites and their contribution to non-enzymatic tolerance mechanisms [72]. The sugars content tends to increase with the plant development, as already reported by Petropoulos et al. [49] and Poli et al. [73] in wild and cultivated Cichorium species. This increase could also affect the taste of the leaves as already reported by Klados and Tzortzakis [69] and Petropoulos et al. [2] in the case of C. spinosum, although the highest increase in our study was observed after the flowering initiation where the leaves are not usually edible. However, in species with a bitter taste such as *C. raphanina* subsp. *mixta*, a high sugars content may not affect the overall flavor of the edible product, since bitterness is highly associated with guaianolides and sesquiterpene lactones which are usually present in *Centaurea* species [74,75]. 

#### 2.2.4. Organic Acids Content

Regarding the organic acids content, the main detected compounds were oxalic and malic acid, followed by citric and ascorbic acid, whereas fumaric acid was detected in very low amounts or only in traces (Table 5). Similar compounds were identified in a previous report by our team [40], even though in lower amounts than the present study. These differences could be mostly attributed to the different growing conditions, as already described in the case of the sugars content (see Section 2.2.3), especially light conditions and temperatures [14,76], since the organic acids content in our study is high even in the control treatments of both harvests, although reducing trends were observed with increasing salinity in both harvests. It could also be suggested that the altitude of the growing location may be a critical factor for the oxalic acid content, since the tested genetic material (seeds) was collected from high altitudes (1300 m above sea level (asl); [40]) and then cultivated from seeds at lower ones (54 m asl), indicating possible adaptation mechanisms. Considering the differences between wild and cultivated plants observed by Petropoulos et al. [40], another possible explanation for the increased content of oxalic acid found in our study could be the high nitrogen availability as well as the nitrogen form, which according to Zhang et al. [77] may affect oxalic acid accumulation. The increased content of oxalic acid, especially in the first harvest where collected leaves are destined for edible use, needs further consideration for safe consumption since the set limit for daily consumption (<5 g) could be exceeded in the case of consumption of 454–474 g fw of leaves or of combined intake along with other oxalic acid dietary sources [20,40]. Moreover, a decreased content of the oxalic, malic and total organic acids contents was recorded in the second harvest of our study, which indicates the role of the developmental stage on organic acids’ biosynthesis and their utilization in biomass production and their allocation in flower and seed formation [76]. The overall low content of ascorbic acid observed in our study could also partly explain the high oxalic acid content since ascorbic acid is a precursor in oxalic acid biosynthesis [78]. 

#### 2.2.5. Fatty Acids Composition

Regarding the fatty acids composition, 18 fatty acids were detected in all the studied samples (Table 6), a finding which is in accordance with the recent report of Petropoulos et al. [40], who tested the same species. Moreover, similarly to that study [40] and studies with other *Centaurea* species [37,79,80], α-linolenic, linoleic and palmitic acid were the richest fatty acids, although a different profile of individual fatty acids was observed which could be due to the effect of growing conditions, as already reported by Nemzer et al. [47] and Petropoulos et al. [14,49]. Moreover, in our study, the α-linolenic content was the highest in the S2 treatment of the first harvest, whereas the lowest content was recorded in the same salinity treatment of the second harvest. Increasing salinity did not have an impact on the linoleic acid content in the first harvest, although it was slightly increased in the second one, whereas the palmitic acid content showed a varied response to salinity levels in the first and second harvest. The increased content of the main fatty acids under saline conditions in the first harvest could be associated with the increased tocopherols content (see Table 3) which can protect lipids from oxidative degradation [14,81,82]. However, apart from the individual fatty acids content, the ratios of PUFA/SFA and n6/n3 fatty acids also show valuable information regarding the nutritional value of the species, where it seems that moderate and high salinity (S1 and S2) resulted in PUFA/SFA and n6/n3 ratios associated with better quality in regards to the fatty acids composition compared with the rest of the treatments [40,83]. Wild edible greens are considered a good source of health beneficial compounds such as *n*-3 fatty acids, therefore, the suggestion of simple cultivation practices that may improve their nutritional value is of major importance for the domestication of these species and their inclusion in human diet on a regular basis. 

#### 2.2.6. Phenolic Compounds Composition

Table 7 presents the data of the chromatographic characteristics (retention time, λ_max_, pseudomolecular ion, major fragment ions in MS^2^) and tentative identification of the individual phenolic compounds present in the studied *Centaurea* samples. Results of the corresponding quantification are presented in Table 8. The characterization of phenolic compounds has been extensively studied in *Centaurea* samples by other authors [18,84]. However, the identification present herein follows the previously described compounds by Petropoulos et al. [40] in samples of the same *Centaurea* species. In the mentioned work, 12 compounds were tentatively identified: five flavonols, two flavones, and five flavanones *O*-glycosylated [40]. The most abundant compounds belonged to the flavanone group which accounted for 83%–92% of the total phenolic compounds detected. The only exception was recorded in the S2 treatment of the first harvest where flavonols were the major detected compounds (Table 7). Pinocembrim neohesperidoside (peak 9) was detected in high amounts in all the tested samples, except for the case of the S2 treatment of the first harvest where only traces were recorded. Other important flavanones were peaks 10, 11 and 12 (pinocembrim acetylarabirosyl glucoside and pinocembrim acetyl neohesperidoside isomer I and II, respectively). Similar results were reported in the study of Petropoulos et al. [40], where the various phenolic compounds classes and the total phenolics content of the cultivated *C. raphanina* subsp. *mixta* plants were within the same range as in our study. Moreover, the highest content of the total phenolic compounds was recorded in the control treatment of the second harvest, mostly due to the abundance of peaks 10, 11 and 12. The same compounds were responsible for the high total phenolic compounds content in the salinity treatments of the same harvest, while the overall total phenolic compounds and total flavanones contents were richer in the second harvest regardless of the salinity treatment. Similarly to our study, the phenolic compounds content increased with the gradual development of *Cichorium spinosum* plants [49], whereas high salinity increased the flavonoids content in the same species (*C. spinosum*) without, however, affecting the total phenolic compounds content [2]. In other studies where the cultivation of wild edible greens was tested, the phenolic compounds content was afffected by successive harvestings and the growing conditions [14,85], or the harvested plant part [8]. Moreover, genotype and stress severity play a crucial role in a plant’s response to high salinity, since the phenolic compounds biosynthesis may increase and act as an intrinsic protective mechanism against stress oxidation in tolerant genotypes [86,87,88] or being disrupted, and decrease in mildly tolerant or in sensitive species subjected to severe stress [89,90]. Moreover, not only phenolic compounds but also other compounds such tocopherols may contribute to the antioxidant mechanisms of plants under stress conditions as already indicated in Section 2.2.2, Table 3 and previous literature reports [91,92,93]. Considering that leaves of the second harvest are not edible, the results of the present study show the potential uses of leaves for other purposes except for culinary ones that could improve the added value of the final product. 

### 2.3. Bioactivity

#### 2.3.1. Antioxidant Activity

The results of the antioxidant capacity estimated with the OxHLIA and TBARS assays showed a varied response to salinity and harvesting time (Table 9). The highest activity was recorded in the control samples of the first and second harvest for the OxHLIA and TBARS assays, respectively. Similarly, in the study of Petropoulos et al. [40], the extracts of the leaves harvested from the cultivated and wild plants also showed a varied response to the same assays. This finding shows a possible involvement of tocopherols, ascorbic acid and phenolic compounds in the antioxidant mechanism of the species, depending on the growth stage and growing conditions, while the varied response observed in this study is in concordance with the literature reports where different assays are tested [29,94,95,96]. For example, tocopherols may inhibit lipid peroxidation and a high content of these compounds results in a high antioxidant activity when a TBARS assay is implemented [94], as was the case in our study. The antioxidant activity of various Centaurea species as well as of C. raphanina subsp. mixta is well reported so far [30,37,40,80,97], showing the great potential of using these natural matrices as functional ingredients in food products. Moreover, the present findings highlight the prospects of using C. raphanina subsp. mixta in nutraceuticals, considering the high antioxidant capacity of the species at the second harvest when the leaves are not used as edible products. 

#### 2.3.2. Cytotoxic Effects

The in vitro cytotoxic effects of the hydroethanolic extracts obtained from *C. raphanina* subsp *mixta* leaves against non-tumor (PLP2: porcine liver primary culture) and tumor (HeLa: cervical carcinoma; HepG2: hepatocellular carcinoma; MCF-7: breast carcinoma and NCI-H460: non-small cell lung cancer) cell lines are presented in Table 10. The results showed no toxicity against the PLP2 and HeLa cell lines, whereas a moderate toxicity was observed against HepG2 (S1 treatment of the first harvest), MCF-7 and NCI-H460 (S2 treatment of the second harvest). These findings are in contrast with those of Petropoulos et al. [40], who reported mild toxicity against the PLP2 and HeLa cell lines, while similarly to our study, they reported an in vitro antiproliferative activity against the HepG2, MCF-7 and NCI-H460 cell lines. Recently, Mikropoulou et al. [45] reported the efficacy of decoctions obtained from leaves of *C. raphanina* against A5 metastatic spindle carcinoma cell lines, while they suggested a mild antiproliferative activity against C5N-immortalized keratinocyte cell lines. Moreover, in the study of Lockowandt et al. [29] where the cytotoxic effects of *C. cyanus* were evaluated, the authors did not report any toxic effects. However, several pharmacological studies have suggested that the cytotoxic effects and the biological activities of various *Centaurea* species are associated with their content in sesquiterpene lactones and flavonoids [25,31,98,99], although the solvents used for the acquisition of extracts may affect the phytochemicals composition in the extracts and therefore their in vitro cytotoxicity [32,100]. Therefore, any cultivation practices and processing of samples that may regulate the phytochemicals content of plants could also affect the bioactive properties of the obtained extracts.

#### 2.3.3. Antimicrobial Properties

The antimicrobial effects of the hydroethanolic extracts obtained from *C. raphanina* subsp. *mixta* leaves are presented in Table 11 and Table 12. All the tested extracts showed a low activity against the tested bacteria when compared with the positive controls (streptomycin and ampicillin) (Table 11). Regardless of that, the most promising results were recorded against *Staphylococcus aureus*, *Bacillus cereus* and *Escherichia coli*, where the lowest minimal inhibition concentration (MIC) and minimal bactericidal concentration (MBC) values were recorded in specific extracts. In particular, the lowest MIC and MBC values against *S. aureus* were recorded in the S1 treatment of the first harvest, while for *B. cereus*, the extracts of plants from the S1 (first harvest) and S2 (second harvest) treatments were the most effective. In the case of *E. coli*, all the tested extracts showed similar effectiveness, except for the case of the control treatment of the first harvest, where a lower activity was observed. Regarding the effectiveness against *Listeria monocytogenes*, *Salmonella typhimurium* and *Enterobacter cloacae*, none of the extracts showed significant activities. Similar results were reported by Petropoulos et al. [40], who concluded that extracts from the wild and cultivated *C. raphanina* subsp. *mixta* leaves were more effective against the abovementioned bacteria. The same bacteria were susceptible to extracts from other *Centaurea* species, while these activities were associated mainly with the presence of sesquiterpene lactones which are very common in that genus [28,34,101]. Considering that in our study pinocembrin and its derivatives were the most abundant polyphenols, it could be assumed that the observed antimicrobial properties are responsible for the bioactive properties of the species. However, further research is needed to test the isolated compounds since in natural matrices synergistic or antagonistic effects may conceal the activities of specific compounds [102,103]. 

Regarding the antifungal properties, most of the extracts had an MIC and minimal fungicidal concentration (MFC) similar to the tested positive controls (bifonazole, ketoconazole) (Table 12). Moreover, extracts from the S1 and S2 treatments of both harvests were more effective (lower MIC values) than bifonazole against *Trichoderma viride*. In the study of Panagouleas et al. [39], the antifungal properties of *C. raphanina* subsp. *mixta* are mostly attributed to the presence of cnicin, a sesquiterpene lactone which, despite its low content, was effective against nine fungi tested including *Aspergillus niger, A. versicolor*, *Penicillium funiculosum* and *Trichoderma viride*, which were also tested in our study. In the same study, it was reported that other compounds such as flavonoids may also contribute to the antifungal activities of the species, a result which is in accordance with our results as well as with the study of Mikropoulou et al. [45], who associated the bioactive properties of the species with the presence of pinocembrin and its derivatives. 

## 3. Materials and Methods 

### 3.1. Plant Material and Growing Conditions

The plant material (seeds) of *C. raphanina* subsp. *mixta* (DC.) Runemark was collected from wild plants at Mainalo Mountain (37.62 N, 22.10 E, altitude: 1194 m above sea level, Arcadia prefecture, Greek) in April 2017, as previously described by the authors [40]. Seeds were put on 5 October 2017 in seed trays filled with peat. Young seedlings were transplanted at the stage of 3 leaves on 11 January 2018 in 2 L plastic pots (one plant per pot) filled with peat (Klassman-Deilmann KTS2) and perlite (2:1; *v*/*v*). After transplantation, seedlings were fertigated with a nutrient solution that contained 300 ppm of N-P-K (control solution; C) by using a water soluble complex fertilizer (Atlas 20-20-20 + TE; [40]). Each pot received 150–200 mL of the control nutrient solution until the plant establishment (2 weeks after transplantation), while after that time period, plants were subjected to salinity stress through the addition of two nutrient solutions: (a) S1 with electrical conductivity (EC) of 4 dS/m and (b) S2 with EC of 6 dS/m. The S1 and S2 solutions were prepared by adding the adequate amount of NaCl in the control solution until the desired level of EC was reached [2]. Fifteen pots were used for each treatment (C, S1 and S2) and 45 pots in total, which were arranged according to the completely randomized design (CRB) in an unheated plastic greenhouse at the University of Thessaly (Volos), Greece.

**Figure 1:**
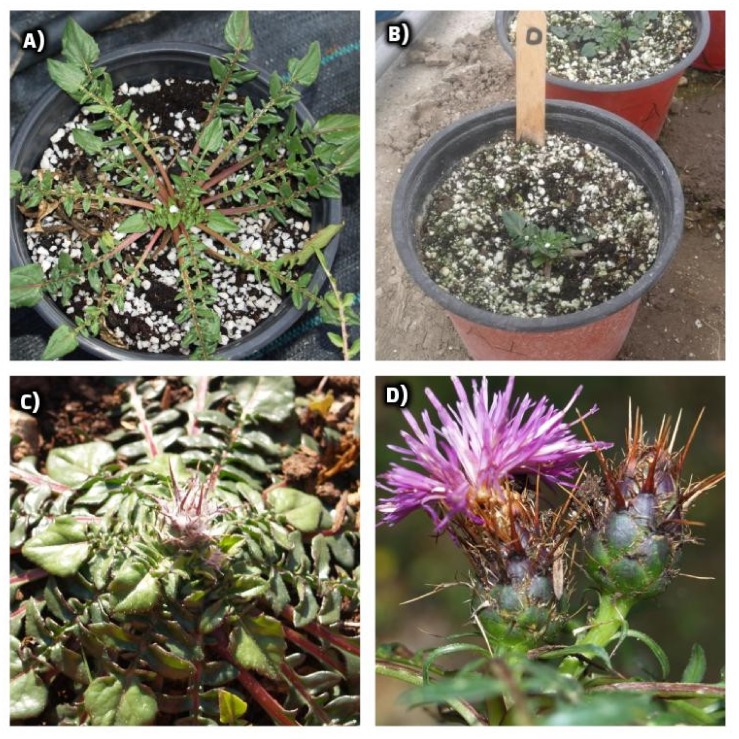
The various developmental phases of *Centaurea raphanina*: (**A**) plant before the first harvest; (**B**) plant after the first harvest; (**C**) plant before the second harvest and when the flower initiation occurred; and (**D**) plants at the flowering stage (full blossom). Photo credits: Spyridon A. Petropoulos.

Plants were harvested twice, namely on 9 March 2018 (first harvest) and on 19 April 2018 (second harvest) when the rosettes of the leaves had a marketable size. The plants of the first harvest were used for the plant growth measurements (fresh weight and moisture content of leaves, rosette diameter, number and thickness of leaves), whereas those of the second harvest were not used for these measurements due to the flowering initiation, which made the leaves unmarketable due to their hard texture. Leaves of the second harvest were collected at the flower initiation and before the flower stalk elongation and the opening of the buds. The various developmental stages of the plants are presented in Figure 1. Leaves of both harvests were used for the chemical analyses, since we aimed to study the effect of prolonged salinity stress on the chemical composition of the leaves and evaluate their potential use not only as edible greens but also for pharmaceutical purposes. For these analyses, fresh samples of leaves were stored in food bags at −20 °C until lyophilization [40]. 

### 3.2. Standards and Reagents

Acetonitrile (99.9%), ethyl acetate (99.8%) and *n*-hexane (95%) were of HPLC grade and acquired from Fisher Scientific (Lisbon, Portugal). Individual compounds were of HPLC or GC grade, and the fatty acids methyl ester (FAME) reference standard mixture 37 (standard 47885-U), individual organic acids and sugars standards were acquired from Sigma-Aldrich (St. Louis, MO, USA). Racemic tocol and tocopherols isoforms were acquired from Matreya, Pleasant Gap, (Pennsyivania, PA, USA). The standards trolox, streptomycin, ampicillin, ketoconazole, bifonazole, ellipticine and sulforhodamine B were acquired from Sigma-Aldrich, (St Louis, MO, USA). The phenolic compounds commercial standards were obtained from Extrasynthèse S.A. (Genay, France) and the human tumour cell lines were acquired from Leibniz-Institute DSMZ (Braunschweig, Germany). Other chemicals and solvents were of analytical grade purity and obtained from common suppliers. A Milli-Q water purification system (TGI Pure Water Systems, Greenville, SC, USA) was used to treat the water.

### 3.3. Chemical Analyses Assays

#### 3.3.1. Proximate Analysis and Energetic Value

The contents of moisture, fat, protein, ash, carbohydrates and energy were estimated according to the Association of Official Analytical Chemists’ (AOAC) procedures [104]. The total carbohydrates were calculated by difference: total carbohydrates (g/100 g fresh weight (fw)) = 100 – (g moisture + g fat + g ash + g proteins), and the total energy was calculated according to the following equation: energy (kcal/100 g fw) = 4 × (g proteins + g carbohydrates) + 9 × (g fat) [105].

#### 3.3.2. Tocopherols

The tocopherols were determined in dried plant material following a method previously described by the authors [105], by HPLC coupled to a fluorescence detector (FP-2020; Jasco, Pfungstadt, Germany) programmed for excitation at 290 nm and emission at 330 nm, using the IS (tocol, 50 mg/mL) method for quantification. The results were presented as mg/100 g fw.

#### 3.3.3. Free Sugars

The dried plant materials were evaluated regarding the sugar content and were determined following a procedure previously optimized by the authors [105], using high-performance liquid chromatography (HPLC) coupled to a refraction index detector (RI). Sugars standards were used for the identification by a chromatographic comparison and the internal standard (melezitose) method was used. The results were presented as g/100 g fw.

#### 3.3.4. Organic Acids

The organic acids were determined in dried plant material and analyzed using UFLC (ultra-fast liquid chromatography; Shimadzu 20A series, Kyoto, Japan) and a photo-diode array detector, as previously optimized and described by the authors [106]. The results were presented as mg/100 g fw.

#### 3.2.5. Fatty Acids

The fatty acids were analyzed after a transesterification procedure and determined using a GC-FID (gas-liquid chromatography with flame ionization detection) equipment with a capillary column, as described previously [105]. The results were presented as a relative percentage of each fatty acid.

#### 3.2.6. Phenolic Compounds

The phenolic compounds and bioactive properties were executed in hydroethanolic extracts by stirring 1 g of the dried plant material with 30 mL of ethanol–water (80:20, *v*/*v*, at 25 °C) for 60 min [107]. The obtained extracts were filtered through Whatman paper No. 4 filters and the residue was re-extracted with the addition of 30 mL of the hydroethanolic solution and filtered as above. The extracts obtained were combined and then evaporated under reduced pressure (Büchi R-210, rotary evaporator, Flawil, Switzerland) until the ethanol was completely removed. After evaporation, the aqueous phase was frozen and lyophilized (FeeeZone 4.5, Labconco, Kansas City, MO, USA).

Phenolic compounds were determined in the freeze-dried power hydroethanolic extracts prepared, re-dissolved in ethanol–water (80:20, *v*/*v*) to a final concentration of 10 mg/mL and filtered through a 0.22 μm disposable filter disk. The compounds were evaluated using a Dionex Ultimate 3000 ultra-performance liquid chromatography (UPLC) system equipped with a quaternary pump and a diode array coupled in-series to an electrospray ionization mass spectrometry detector (LC-DAD-ESI/MS^n^) [107]. The identification of the individual phenolic compounds was performed by comparing the retention times, UV–visible spectra and the MS fragmentation patterns of the detected compounds with those of authentic standards, and data available from the literature were also used. The quantification was based on the calibration curves of authentic standards. The results were presented as mg/g of plant fw.

### 3.4. Antioxidant Activity

The antioxidant activity was evaluated by applying two cell-based assays: the oxidative haemolysis (OxHLIA) and the thiobarbituric acid reactive substances (TBARS) formation inhibition assays previously described by Lockowandt et al. [29], using the above-prepared hydroethanolic extracts. The used positive control was Trolox. 

#### 3.4.1. OxHLIA Assay

The antihaemolytic activity was determined by the oxidative haemolysis inhibition assay (OxHLIA) [29]. The results were expressed as IC_50_ values, which is the extract concentration (µg/mL) required to inhibit oxidative haemolysis of 50% of the erythrocytes for *Δ*t for 60 min.

#### 3.4.2. TBARS Assay

For the TBARS assay, brain tissues from *Sus scrofa* were dissected and homogenized with a Tris-HCl buffer (20 mM, pH 7.4) to obtain a homogenate (1:2; *w*/*v*) of the brain tissue and was then centrifuged for 10 min at 3000 *g*. The extract samples (0.2 mL) were incubated at 37 °C for 1 h with the porcine brain supernatant (1:2, *w*/*v*; 0.1 mL), FeSO_4_ (10 μM; 0.1 mL) and ascorbic acid (0.1 mM; 0.1 mL). Then, tri-chloroacetic (28% *w*/*v*, 0.5 mL) and thiobarbituric (TBA, 2%, *w*/*v*, 0.38 mL) acids were added and the mixture was heated at 80 °C for 20 min and centrifuged for 5 min at 3000 [29]. The results were presented as EC_50_ values, which is the extract concentration (μg/mL) that provides 50% of the antioxidant activity.

### 3.5. Hepatotoxicity and Cytotoxicity Assays

The hepatotoxicity was evaluated using the sulforhodamine B assay. Briefly, primary cell cultures (PLP2) were prepared from porcine liver and tested with different concentrations of the above-prepared hydroethanolic extracts, ranging from 400 to 6.5 μg/mL. The anti-proliferative capacity of the extracts was also evaluated by using four human tumor cell lines, namely HeLa (cervical carcinoma), HepG2 (hepatocellular carcinoma), MCF-7 (breast adenocarcinoma) and NCI-H460 (non-small cell lung cancer) [108]. In both the hepatotoxicity and cytotoxicity assays, ellipticine was used as the positive control and the results were presented as GI_50_ values (μg/mL), which correspond to the extract concentration that inhibits cell growth by 50%.

### 3.6. Antimicrobial Properties

The potential antimicrobial activity was measured in the hydroethanolic extracts, and Gram-positive bacteria *Staphylococcus aureus* (ATCC 6538), *Bacillus cereus* (food isolate), *Listeria monocytogenes* (NCTC 7973), as well as the Gram-negative bacteria *Escherichia coli* (ATCC 25922), *Salmonella typhimurium* (ATCC 13311) and *Enterobacter cloacae* (ATCC 35030) were used. For the antifungal assays, six micromycetes were used: *Aspergillus fumigatus* (human isolate), *Aspergillus niger* (ATCC 6275), *Aspergillus versicolor* (ATCC 11730), *Penicillium funiculosum* (ATCC 36839), *Trichoderma viride* (IAM 5061) and *Penicillium verrucosum* var. *cyclopium* (food isolate). For the antimicrobial properties of the hydroethanolic extracts, the microdilution method was used [109]. The results were presented as the concentrations that resulted in the complete inhibition of the bacterial growth (MIC, minimal inhibition concentration), through the colorimetric microbial viability assay, as well as MBC and MFC values (minimal bactericidal concentration and minimal fungicidal concentration, respectively). The used positive controls were streptomycin, ampicillin, ketoconazole and bifonazole, whereas the negative control was 5% DMSO.

### 3.7. Statistical Analysis

The chemical composition and bioactivity analyses were carried out on three samples for each treatment and all the assays were performed in triplicate. Data were checked with Shapiro–Wilk normality test to ensure they followed the normal distribution before the analysis. For the analysis of the data, the Statgraphics 5.1.plus (Statpoint Technologies, Inc., VA, USA) software was used. Data were evaluated by a two-way ANOVA for the effect of salinity and harvesting time, while means comparisons were performed with the Duncan’s multiple range test (*p* = 0.05).

## 4. Conclusions

The results of our study show the potential of cultivating *C. raphanina* subsp. *mixta* plants under salinity conditions, since the mild salinity stress (4.0 dS/m) tested in this report did not severely decrease plant growth while at the same time improved the specific compositional parameters that could increase the quality and the bioactive properties of the final product. Moreover, harvest time is also a cost-effective cultivation practice that can improve the added value of the species, since by applying successive harvests, we may obtain edible leaves from the first harvest, while the leaves of the second harvest could be used for nutraceutical and pharmaceutical purposes as well as antimicrobial agents in food products. This finding is justified by the lower oxalic acid content and the increased tocopherols and phenolic compounds contents, which are highly associated with the bioactive potential of the species. Therefore, the commercial growing of *C. raphanina* subsp. *mixta* could be suggested in mildly salt-affected soils as well as in regions where brackish water is available for agricultural uses.

## Figures and Tables

**Table 1:** Plant growth parameters of *Centaurea raphanina* subsp. *mixta* leaves in relation to harvesting time (first and second harvest) and salinity level (control, S1 and S2) (mean ± SD; *n* = 15).

Harvesting Time *	Salinity Level	Fresh Weight (g Per Plant)	Rosette Diameter (cm)	Number of Leaves	Leaf Thickness (mm)
1st	C	11.1 ± 0.7a	19.8 ± 1.1a	12.5 ± 2.1a	0.75 ± 0.05a
	S1	9.8 ± 1.1b	22.5 ± 2.3a	12.2 ± 2.8a	0.7 ± 0.1a
	S2	8.05 ± 1.17c	19.8 ± 1.7a	11.95 ± 1.78a	0.74 ± 0.08a

* Harvesting time: first (9 March 2018); results from the second harvest (19 April 2018) are not available due to early anthesis of plants; salinity level—C: control treatment (2.0 dS/m), S1 (4.0 dS/m) and S2 (6.0 dS/m). Different Latin letters in the same column indicate significant differences between the means according to the Duncan multiple range test at *p* = 0.05.

**Table 2:** Nutritional value (g/100 g fresh weight (fw)) and energetic value (kcal/100 g fw) of *Centaurea raphanina* subsp. *mixta* leaves in relation to harvesting time (H) and salinity level (S) (mean ± SD, *n* = 3).

H *	S	Moisture	Fat	Proteins	Ash	Carbohydrates	Energy
1st	C	88.9 ± 0.6d	0.31 ± 0.01d	3.82 ± 0.01b	1.76 ± 0.04e	5.22 ± 0.02c	38.9 ± 0.1b
	S1	88.8 ± 0.2d	0.33 ± 0.03c	3.71 ± 0.01c	1.86 ± 0.07d	5.3 ± 0.1b	39.0 ± 0.1b
	S2	87.6 ± 0.4e	0.45 ± 0.01a	3.9 ± 0.2a	2.19 ± 0.07b	5.9 ± 0.1a	43.2 ± 0.2a
2nd	C	90.6 ± 0.5a	0.30 ± 0.01d	2.67 ± 0.01f	1.77 ± 0.02e	4.68 ± 0.01f	32.1 ± 0.1d
	S1	89.8 ± 0.2b	0.41 ± 0.02b	2.82 ± 0.02d	2.02 ± 0.01c	4.93 ± 0.02e	34.7 ± 0.1c
	S2	89.3 ± 0.7c	0.40 ± 0.01b	2.75 ± 0.02e	2.54 ± 0.01a	5.04 ± 0.02d	34.7 ± 0.1c

* H: harvesting time—first (9 March 2018) and second (19 April 2018) harvesting; S: salinity level—C: control treatment (2.0 dS/m), S1 (4.0 dS/m) and S2 (6.0 dS/m). Different Latin letters in the same column indicate significant differences between the means according to the Duncan multiple range test at *p* = 0.05.

**Table 3:** Composition in tocopherols (mg/100 g fw) of *Centaurea raphanina* subsp. *mixta* leaves in relation to harvesting time (H) and salinity level (S) (mean ± SD, *n* = 3).

H *	S	α-Tocopherol	γ-Tocopherol	Total Tocopherols
1st	C	0.094 ± 0.001e	0.025 ± 0.001e	0.120 ± 0.001e
	S1	0.404 ± 0.006c	0.072 ± 0.001a	0.480 ± 0.007c
	S2	0.368 ± 0.007d	0.055 ± 0.001b	0.430 ± 0.007d
2nd	C	0.68 ± 0.01a	0.047 ± 0.001c	0.730 ± 0.007a
	S1	0.69 ± 0.01a	0.055 ± 0.001b	0.74 ± 0.01a
	S2	0.559 ± 0.003b	0.036 ± 0.005d	0.600 ± 0.007b

* H: harvesting time—first (9 March 2018) and second (19 April 2018) harvesting; S: salinity level—C: control treatment (2.0 dS/m), S1 (4.0 dS/m) and S2 (6.0 dS/m). Different Latin letters in the same column indicate significant differences between the means according to the Duncan multiple range test at *p* = 0.05.

**Table 4:** Composition in sugars (g/100 g fw) of *Centaurea raphanina* subsp. *mixta* leaves in relation to harvesting time (H) and salinity level (S) (mean ± SD, *n* = 3).

H *	S	Fructose	Glucose	Sucrose	Trehalose	Total Sugars
1st	C	0.23 ± 0.03c	0.206 ± 0.004b	0.155 ± 0.006d	0.167 ± 0.007f	0.76 ± 0.05c
	S1	0.14 ± 0.02f	0.205 ± 0.003b	0.139 ± 0.003e	0.190 ± 0.005e	0.67 ± 0.01e
	S2	0.25 ± 0.03b	0.216 ± 0.006a	0.139 ± 0.008e	0.201 ± 0.007d	0.80 ± 0.04b
2nd	C	0.16 ± 0.02e	0.083 ± 0.001e	0.285 ± 0.007a	0.211 ± 0.004c	0.74 ± 0.03d
	S1	0.170 ± 0.001d	0.122 ± 0.001c	0.244 ± 0.003c	0.271 ± 0.001b	0.810 ± 0.002b
	S2	0.50 ± 0.03a	0.106 ± 0.009d	0.261 ± 0.007b	0.322 ± 0.002a	1.19 ± 0.03a

* H: harvesting time—first (9 March 2018) and second (19 April 2018) harvesting; S: salinity level—C: control treatment (2.0 dS/m), S1 (4.0 dS/m) and S2 (6.0 dS/m). Different Latin letters in the same column indicate significant differences between the means according to the Duncan multiple range test at *p* = 0.05.

**Table 5:** Composition in organic acids (mg/100 g fw) of *Centaurea raphanina* subsp. *mixta* leaves in relation to harvesting time (H) and salinity level (S) (mean ± SD, *n* = 3).

H *	S	Oxalic Acid	Malic Acid	Ascorbic Acid	Citric Acid	Fumaric Acid	Total Organic Acids
1st	C	1060 ± 2c	718 ± 1a	0.28 ± 0.01b	431 ± 9f	tr	2210 ± 7a
	S1	1101 ± 2a	289 ± 3e	0.28 ± 0.01b	478 ± 5e	tr	1868 ± 10c
	S2	1072 ± 4b	299 ± 5d	0.210 ± 0.006c	581 ± 5a	tr	1957 ± 8b
2nd	C	877 ± 2e	447 ± 2b	0.60 ± 0.02a	492 ± 3d	0.08 ± 0.02	1817 ± 3d
	S	895 ± 3d	316 ± 2c	0.08 ± 0.02d	568 ± 3b	tr	1779 ± 2e
	S2	793 ± 4f	259 ± 1f	tr	544.1 ± 0.1c	tr	1596 ± 6f

Tr—traces. * H: harvesting time—first (9 March 2018) and second (19 April 2018) harvesting; S: salinity level—C: control treatment (2.0 dS/m), S1 (4.0 dS/m) and S2 (6.0 dS/m). Different Latin letters in the same column indicate significant differences between the means according to the Duncan multiple range test at *p* = 0.05.

**Table 6:** Fatty acids composition (relative %) of *Centaurea raphanina* subsp. *mixta* leaves in relation to harvesting time and salinity level (mean ± SD, *n* = 3).

	1st Harvest *			2nd Harvest		
Fatty Acid	Control	S1	S2	Control	S1	S2
C8:0	0.066 ± 0.004c	0.068 ± 0.002	0.096 ± 0.004a	0.067 ± 0.004c	0.09 ± 0.02b	0.091 ± 0.006b
C10:0	0.053 ± 0.005d	0.057 ± 0.001d	0.089 ± 0.004b	0.083 ± 0.008bc	0.08 ± 0.01c	0.109 ± 0.005a
C11:0	0.139 ± 0.009d	0.216 ± 0.001c	0.37 ± 0.02a	0.30 ± 0.03b	0.25 ± 0.04c	0.36 ± 0.03a
C12:0	0.239 ± 0.002d	0.31 ± 0.03c	0.095 ± 0.002e	0.25 ± 0.02d	0.462 ± 0.005a	0.43 ± 0.01b
C14:0	2.6 ± 0.2d	1.08 ± 0.01e	1.06 ± 0.05e	5.71 ± 0.06c	12.0 ± 0.1a	9.3 ± 0.1b
C14:1	0.033 ± 0.001d	0.044 ± 0.002c	0.109 ± 0.001b	0.017 ± 0.001f	0.023 ± 0.001e	0.234 ± 0.006a
C15:0	0.26 ± 0.02a	0.25 ± 0.01ab	0.185 ± 0.003d	0.215 ± 0.001c	0.213 ± 0.001c	0.238 ± 0.008b
C16:0	21.7 ± 0.1b	19.2 ± 0.9d	16.95 ± 0.04e	20.3 ± 0.2c	21.7 ± 0.2b	22.84 ± 0.02a
C17:0	0.283 ± 0.006bc	0.225 ± 0.003d	0.217 ± 0.004e	0.28 ± 0.01c	0.287 ± 0.006b	0.347 ± 0.005a
C18:0	2.4 ± 0.2b	2.0 ± 0.1c	1.65 ± 0.04d	1.97 ± 0.05c	2.07 ± 0.01c	2.62 ± 0.01a
C18:1n9c	2.05 ± 0.05c	1.51 ± 0.06e	2.1 ± 0.1c	1.92 ± 0.02d	2.24 ± 0.01b	2.73 ± 0.01a
C18:2n6c	26.3 ± 0.2a	26.5 ± 0.6a	26.4 ± 0.2a	23.7 ± 0.1c	24.47 ± 0.03b	24.36 ± 0.05b
C18:3n3	40.7 ± 0.2d	45.4 ± 0.1b	48.37 ± 0.07a	43.1 ± 0.1c	33.86 ± 0.08e	33.35 ± 0.05f
C20:0	0.43 ± 0.04b	0.35 ± 0.02d	0.317 ± 0.003e	0.365 ± 0.001d	0.399 ± 0.009c	0.53 ± 0.02a
C21:0	0.149 ± 0.009b	0.168 ± 0.004a	0.167 ± 0.004a	0.084 ± 0.003d	0.070 ± 0.008e	0.13 ± 0.01c
C22:0	0.68 ± 0.02b	0.68 ± 0.06b	0.575 ± 0.009d	0.613 ± 0.005cd	0.63 ± 0.03c	0.98 ± 0.05a
C23:0	0.39 ± 0.01a	0.30 ± 0.01b	0.181 ± 0.009e	0.25 ± 0.01d	0.281 ± 0.001c	0.284 ± 0.001c
C24:0	1.42 ± 0.08b	1.69 ± 0.08a	1.1 ± 0.1c	0.748 ± 0.005e	0.89 ± 0.04d	1.03 ± 0.02c
SFA	30.9 ± 0.4b	26.6 ± 0.5c	23.02 ± 0.01d	31.2 ± 0.1b	39.4 ± 0.1a	39.3 ± 0.1a
MUFA	2.08 ± 0.05c	1.56 ± 0.06e	2.2 ± 0.1b	1.94 ± 0.02d	2.26 ± 0.01b	2.96 ± 0.01a
PUFA	67.0 ± 0.4c	71.9 ± 0.4b	74.8 ± 0.1a	66.8 ± 0.1c	58.3 ± 0.1d	57.7 ± 0.1e
PUFA/SFA	2.17 ± 0.4c	2.7 ± 0.4b	3.25 ± 0.05a	2.1 ± 0.1c	1.5 ± 0.1d	1.47 ± 0.1d
n6/n3	0.65 ± 0.18b	0.58 ± 0.15c	0.55 ± 0.14d	0.55 ± 0.11d	0.72 ± 0.45a	0.73 ± 0.04a

* H: harvesting time—first (9 March 2018) and second (19 April 2018) harvesting; salinity level—C: control treatment (2.0 dS/m), S1 (4.0 dS/m) and S2 (6.0 dS/m). Caprylic acid (C8:0); capric acid (C10:0); undecylic acid (C11:0); lauric acid (C12:0); myristic acid (C14:0); myristoleic acid (C14:1); pentadecylic acid (C15:0); palmitic acid (C16:0); margaric acid (C17:0); stearic acid (C18:0); oleic acid (C18:1n9); linoleic acid (C18:2n6c); α-linolenic acid (C18:3n3); arachidic acid (C20:0); heneicosylic acid (C21:0); behenic acid (C22:0); tricosylic acid (C23:0); lignoceric acid (C24:0); SFA: saturated fatty acids; MUFA: monounsaturated fatty acids; PUFA: polyunsaturated fatty acids; n6/n3: omega-6/omega-3 fatty acids. Different Latin letters in the same row indicate significant differences between the means according to the Duncan multiple range test at *p* = 0.05.

**Table 7:** Retention time (Rt), wavelengths of maximum absorption in the visible region (λ_max_), mass spectral data and tentative identification of the phenolic compounds present in the hydroethanolic extracts of *Centaurea raphanina* subsp. *mixta* leaves.

Peak	Rt (min)	λ_max_ (nm)	[M−H]^−^ (*m*/*z*)	MS^2^ (*m*/*z*)	Tentative Identification
1	14.16	349	493	317(100)	Myricetin-*O*-glucoside
2	18.1	344	477	301(100)	Quercetin-3-*O*-glucoside
3	18.63	334	461	285(100)	Kaempherol-*O*-glucoronide
4	20.4	334	579	285(100)	Kaempherol-*O-*hexoside-pentoside
5	22.14	334	563	269(100)	Apigenin-*O*-hexoside-pentoside
6	22.9	334	445	269(100)	Apigenin-*O*-glucoronide
7	25.44	332	665	621(100), 285(45)	Kaempherol-*O*-malonyl-pentoside
8	28.28	286/326	549	429(12), 297(14), 279(5), 255(41)	Pinocembrim arabirosyl glucoside
9	29.47	286/326	563	443(12), 401(5), 297(21), 255(58)	Pinocembrim neohesperidoside
10	31.39	288/328	591	549(30), 429(20), 297(15), 279(5), 255(32)	Pinocembrim acetylarabirosyl glucoside
11	31.79	285/326	605	563(12), 545(5), 443(30), 401(10), 255(40)	Pinocembrim acetyl neohesperidoside isomer I
12	32.14	286/328	605	563(10), 545(5), 443(28), 401(9), 255(39)	Pinocembrim acetyl neohesperidoside isomer II

**Table 8:** Quantification (mg/g of plant fw) of the phenolic compounds present in the hydroethanolic extracts of *Centaurea raphanina* subsp. *mixta* leaves in relation to salinity level and harvesting time (mean ± SD, *n* = 3).

Peaks	1st Harvest *			2nd Harvest		
	C	S1	S2	C2	S1	S2
1	0.074 ± 0.001a	0.071 ± 0.001a	0.073 ± 0.001a	0.062 ± 0.002b	0.057 ± 0.001c	0.063 ± 0.001b
2	0.021 ± 0.001a	0.016 ± 0.001b	0.010 ± 0.002c	0.015 ± 0.001b	0.011 ± 0.001c	0.013 ± 0.001bc
3	0.047 ± 0.001a	0.031 ± 0.001b	0.014 ± 0.001e	0.034 ± 0.001b	0.019 ± 0.001d	0.029 ± 0.001c
4	0.024 ± 0.001a	0.02 ± 0.004b	0.014 ± 0.001c	0.018 ± 0.001b	0.012 ± 0.001c	0.019 ± 0.001b
5	0.022 ± 0.001a	0.019 ± 0.001b	0.013 ± 0.001c	0.02 ± 0.01ab	0.013 ± 0.001c	0.019 ± 0.001b
6	0.023 ± 0.001a	0.02 ± 0.001b	0.013 ± 0.001d	0.019 ± 0.001bc	0.012 ± 0.001d	0.017 ± 0.001c
7	0.015 ± 0.001b	0.014 ± 0.001b	0.019 ± 0.001a	0.015 ± 0.001b	0.011 ± 0.001c	0.015 ± 0.001b
8	0.033 ± 0.001d	0.023 ± 0.001e	0.078 ± 0.001a	0.055 ± 0.003b	0.045 ± 0.0002c	0.032 ± 0.001d
9	0.86 ± 0.02a	0.79 ± 0.05b	tr	0.7 ± 0.1c	0.65 ± 0.01d	0.64 ± 0.01d
10	0.041 ± 0.002d	0.024 ± 0.001e	tr	0.156 ± 0.003a	0.091 ± 0.002b	0.049 ± 0.001c
11	0.030 ± 0.001d	0.020 ± 0.001e	0.007 ± 0.001f	0.113 ± 0.004a	0.074 ± 0.004c	0.087 ± 0.004b
12	0.29 ± 0.01d	0.16 ± 0.01e	tr	0.94 ± 0.04a	0.64 ± 0.01c	0.9 ± 0.1b
Tfols	0.181 ± 0.001a	0.152 ± 0.002b	0.133 ± 0.001e	0.144 ± 0.001c	0.124 ± 0.001f	0.139 ± 0.001d
Tflavones	0.045 ± 0.001a	0.039 ± 0.001b	0.025 ± 0.001e	0.039 ± 0.001b	0.034 ± 0.001d	0.036 ± 0.001c
Tflav	1.25 ± 0.03d	1.0 ± 0.1e	0.086 ± 0.001f	1.9 ± 0.1a	1.50 ± 0.02c	1.7 ± 0.1b
TPC	1.48 ± 0.03d	1.2 ± 0.1e	0.245 ± 0.001f	2.1 ± 0.1a	1.63 ± 0.02c	1.9 ± 0.1b

* H: harvesting time—first (9 March 2018) and second (19 April 2018) harvesting; S: salinity level—C: control treatment (2.0 dS/m), S1 (4.0 dS/m) and S2 (6.0 dS/m). Tfols: total flavonols; Tflavones: total flavones; Tflavn: total flavanones; TPC: total phenolic compounds. Tr—traces; nd—not detected. Standard calibration curves used for quantification: apigenin-7-*O*-glucoside (*y* = 10683*x* – 45794, *R²* = 0.996, LOD = 0.10 µg/mL and LOQ = 0.53 µg/mL, peaks 5 and 6); myricetin (*y* = 23287*x* – 581708, *R²* = 0.9988, LOD = 0.23 µg/mL and LOQ = 0.78 µg/mL, peak 1); naringenin (*y* = 18433*x* + 78903, *R²* = 0.9998, LOD = 0.17 µg/mL and LOQ = 0.81 µg/mL, peaks 8, 9, 10, 11, and 12); and quercetin-3-*O*-glucoside (*y* = 34843*x* – 160173, *R²* = 0.9998, LOD = 0.21 µg/mL and LOQ = 0.71 µg/mL, peaks 2, 3, 4, and 7). Different Latin letters within the same row indicate significant differences among the means of the different salinity levels and harvesting times.

**Table 9:** Antioxidant activity of *Centaurea raphanina* subsp. *mixta* leaves’ hydroethanolic extracts in relation to harvesting time and salinity level (mean ± SD, *n* = 3).

H *	S	OxHLIA (IC_50_; µg/mL); Δt = 60 min	TBARS (EC_50_, μg/mL)
1st	C	81 ± 5f	46.1 ± 0.5a
	S1	139 ± 9b	40 ± 2b
	S2	89 ± 3e	40 ± 2b
2nd	C	111 ± 3d	32 ± 2d
	S1	189 ± 2a	34 ± 2c
	S2	116 ± 3c	46 ± 2a

EC_50_: extract concentration corresponding to a 50% of antioxidant activity. Trolox EC_50_ values: 23 ± 0.1 µg/mL (TBARS inhibition) and 19.6 ± 0.1 µg/mL (OxHLIA Δt = 60 min). * H: harvesting time—first (9 March 2018) and second (19 April 2018) harvesting; S: salinity level—C: control treatment (2.0 dS/m), S1 (4.0 dS/m) and S2 (6.0 dS/m). Different Latin letters in the same column indicate significant differences between the means according to the Duncan multiple range test at *p* = 0.05.

**Table 10:** Cytotoxicity and antitumor activity (GI_50_ values μg/mL) of *Centaurea raphanina* subsp. *mixta* leaves’ hydroethanolic extracts in relation to harvesting time and salinity level (mean ± SD, *n* = 3).

		Cytotoxicity to Non-Tumor Cell Lines	Cytotoxicity to Tumor Cell Lines			
H *	S	PLP2 (Porcine Liver Primary Culture)	HeLa (Cervical Carcinoma)	HepG2 (Hepatocellular Carcinoma)	MCF-7 (Breast Carcinoma)	NCI-H460 (Non-Small Cell Lung Cancer)
1st	C	>400	>400	307 ± 13c	>400	297 ± 5b
	S1	>400	>400	225 ± 13d	354 ± 20a	369 ± 29a
	S2	>400	>400	>400	>400	>400
2nd	C	>400	>400	>400	>400	286 ± 1c
	S1	>400	>400	332 ± 31b	>400	>400
	S2	>400	>400	388 ± 3a	297 ± 17b	280 ± 20d

GI50 values correspond to the sample concentration responsible for the 50% inhibition of growth in tumor cells or in a primary culture of liver cells—PLP2. GI50 values for Ellipticine (positive control): 1.21 ± 0.02 μg/mL (MCF-7), 1.03 ± 0.09 μg/mL (NCI-H460), 0.9 ± 0.1 μg/mL (HeLa), 1.10 ± 0.09 μg/mL (HepG2) and 2.3 ± 0.2 μg/mL (PLP2). * H: harvesting time—first (9 March 2018) and second (19 April 2018) harvesting; S: salinity level—C: control treatment (2.0 dS/m), S1 (4.0 dS/m) and S2 (6.0 dS/m). Different Latin letters in the same column indicate significant differences between the means according to the Duncan multiple range test at *p* = 0.05.

**Table 11:** Antibacterial activity (minimal inhibition concentration (MIC) and minimal bactericidal concentration (MBC) mg/mL) of *Centaurea raphanina* subsp. *mixta* leaves’ hydroethanolic extracts in relation to harvesting time and salinity level.

H *	S	MIC/MBC	*S. aureus * (ATCC 11632)	*B. cereus* (Food Isolate)	*L. monocytogenes * (NCTC 7973)	*E. coli* (ATCC 25922)	*S. typhimurium * (ATCC 13311)	*E. cloacae * (ATCC 35030)
1st	C	MIC	1	1	2	1	2	2
		MBC	2	2	4	2	4	4
	S1	MIC	0.5	0.5	2	0.5	2	2
		MBC	1	1	4	1	4	4
	S2	MIC	1	1	2	0.5	1	4
		MBC	2	2	4	1	2	8
2nd	C	MIC	1	1	2	0.5	2	2
		MBC	2	2	4	1	4	4
	S	MIC	1	1	2	0.5	2	2
		MBC	2	2	4	1	4	4
	S2	MIC	1	0.5	2	0.5	2	2
		MBC	2	1	4	1	4	4
Positive controls	Streptomycin	MIC	0.1	0.025	0.15	0.1	0.1	0.025
		MBC	0.2	0.05	0.3	0.2	0.2	0.05
	Ampicillin	MIC	0.1	0.1	0.15	0.15	0.1	0.1
		MBC	0.15	0.15	0.3	0.2	0.2	0.15

* H: harvesting time—first (9 March 2018) and second (19 April 2018) harvesting; S: salinity level—C: control treatment (2.0 dS/m), S1 (4.0 dS/m) and S2 (6.0 dS/m). MIC = minimal inhibition concentration; MBC = minimal bactericidal concentration.

**Table 12:** Antifungal activity of *Centaurea raphanina* subsp. *mixta* leaves’ hydroethanolic extracts in relation to salinity level (control, S1 and S2) and harvesting time (MIC and MFC mg/mL).

H *	S	MIC/MFC	*Aspergillus fumigatus* (ATCC 9197)	*Aspergillus niger* (ATCC 6275)	*Aspergillus versicolor* (ATCC 11730)	*Penicillium funiculosum* (ATCC 36839	*Trichoderma viride* (IAM 5061)	*Penicillium verrucosum var. cyclopium* (Food Isolate)
1st	Control 1st	MIC	0.25	0.25	0.25	0.25	0.25	0.5
		MFC	0.5	0.5	0.5	0.5	0.5	1
	S1 1st	MIC	0.5	0.5	0.25	0.25	0.12	0.25
		MFC	1	1	0.5	0.5	0.25	0.5
	S2 1st	MIC	0.25	0.25	0.25	0.25	0.12	0.25
		MFC	0.5	0.5	0.5	0.5	0.25	0.5
2nd	Control 2nd	MIC	0.5	0.5	0.5	0.25	0.25	0.25
		MFC	1	1	1	0.5	0.5	0.5
	S1 2nd	MIC	0.5	0.5	0.5	0.25	0.12	0.25
		MFC	1	1	1	0.5	0.25	0.5
	S2 2nd	MIC	0.5	0.5	0.5	0.25	0.12	0.25
		MFC	1	1	1	0.5	0.25	0.5
Positive controls	Bifonazole	MIC	0.15	0.15	0.1	0.2	0.15	0.1
		MFC	0.2	0.2	0.2	0.25	0.2	0.2
	Ketoconazole	MIC	0.2	0.2	0.2	0.2	1	0.2
		MFC	0.5	0.5	0.5	0.5	1.5	0.3

* H: harvesting time—first (9 March 2018) and second (19 April 2018) harvesting; S: salinity level—C: control treatment (2.0 dS/m), S1 (4.0 dS/m) and S2 (6.0 dS/m). MIC = minimal inhibition concentration; MBC = minimal bactericidal concentration.

## References

[1] Petropoulos S., Ntatsi G., Levizou E., Barros L., Ferreira I. (2016). Nutritional profile and chemical composition of *Cichorium spinosum* ecotypes. Lwt-Food Sci. Technol..

[2] Petropoulos S., Levizou E., Ntatsi G., Fernandes Â., Petrotos K., Akoumianakis K., Barros L., Ferreira I. (2017). Salinity effect on nutritional value, chemical composition and bioactive compounds content of *Cichorium spinosum* L.. Food Chem..

[3] Zhu J.K. (2001). Plant salt tolerance. Trends Plant Sci..

[4] Hazell P., Poulton C., Wiggins S., Dorward A. (2006). The future of small farms: Synthesis paper. World Dev. Rep..

[5] Petropoulos S.A., Karkanis A., Martins N., Ferreira I.C.F.R. (2018). Edible halophytes of the Mediterranean basin: Potential candidates for novel food products. Trends Food Sci. Technol..

[6] Nebel S., Heinrich M. (2009). Ta chórta: A comparative ethnobotanical-linguistic study of wild food plants in a graecanic area in Calabria, Southern Italy. Econ. Bot..

[7] Pinela J., Carvalho A.M., Ferreira I.C.F.R. (2017). Wild edible plants: Nutritional and toxicological characteristics, retrieval strategies and importance for today ’s society. Food Chem. Toxicol..

[8] Petropoulos S.A., Fernandes Â., Dias M.I., Vasilakoglou I.B., Petrotos K., Barros L., Ferreira I.C.F.R. (2019). Nutritional value, chemical composition and cytotoxic properties of common purslane (*Portulaca oleracea* L.) in relation to harvesting stage and plant part. Antioxidants.

[9] Petropoulos S., Karkanis A., Fernandes Â., Barros L., Ferreira I.C.F.R., Ntatsi G., Petrotos K., Lykas C., Khah E. (2015). Chemical composition and yield of six genotypes of common purslane (*Portulaca oleracea* L.): An alternative source of omega-3 fatty acids. Plant Foods Hum. Nutr..

[10] Luczaj L., Pieroni A., Tardío J., Pardo-De-Santayana M., Sõukand R., Svanberg I., Kalle R. (2012). Wild food plant use in 21st century Europe: The disappearance of old traditions and the search for new cuisines involving wild edibles. Acta Soc. Bot. Pol..

[11] Disciglio G., Tarantino A., Frabboni L., Gagliardi A., Michela M., Tarantino E., Gatta G., Beta L., Miller F., Cichorium L. (2017). Qualitative characterisation of cultivated and wild edible plants: Mineral elements, phenols content and antioxidant capacity. Ital. J. Agron..

[12] Alu’datt M.H., Rababah T., Alhamad M.N., Al-Tawaha A., Al-Tawaha A.R., Gammoh S., Ereifej K.I., Al-Karaki G., Hamasha H.R., Tranchant C.C. (2019). Herbal yield, nutritive composition, phenolic contents and antioxidant activity of purslane (*Portulaca oleracea* L.) grown in different soilless media in a closed system. Ind. Crop. Prod..

[13] Papafilippaki A., Nikolaidis N.P. (2020). Comparative study of wild and cultivated populations of *Cichorium spinosum*: The influence of soil and organic matter addition. Sci. Hortic. (Amst. )..

[14] Petropoulos S., Fernandes Â., Karkanis A., Ntatsi G., Barros L., Ferreira I. (2017). Successive harvesting affects yield, chemical composition and antioxidant activity of *Cichorium spinosum* L.. Food Chem..

[15] Petropoulos S.A., Fernandes Â., Calhelha R.C., Di Gioia F., Kolovou P., Barros L., Ferreira I.C.F.R. (2019). Chemical composition and bioactive properties of *Cichorium spinosum* L. in relation to nitrate/ammonium nitrogen ratio. J. Sci. Food Agric..

[16] Ceccanti C., Landi M., Benvenuti S., Pardossi A., Guidi L. (2018). Mediterranean wild edible plants: Weeds or “new functional crops”?. Molecules.

[17] Petropoulos S., Fernandes A., Barros L., Ferreira I. (2017). A comparison of the phenolic profile and antioxidant activity of different *Cichorium spinosum* L. ecotypes. J. Sci. Food Agric..

[18] Pires T.C.S.P., Dias M.I., Barros L., Calhelha R.C., Alves M.J., Oliveira M.B.P.P., Santos-Buelga C., Ferreira I.C.F.R. (2018). Edible flowers as sources of phenolic compounds with bioactive potential. Food Res. Int..

[19] Pereira C., Barros L., Carvalho A.M., Ferreira I.C.F.R. (2011). Nutritional composition and bioactive properties of commonly consumed wild greens: Potential sources for new trends in modern diets. Food Res. Int..

[20] Guil J.L., Torija M.E., Giménez J.J., Rodríguez-García I., Himénez A. (1996). Oxalic acid and calcium determination in wild edible plants. J. Agric. Food Chem..

[21] Guil J.L., Rodríguez-García I., Torija E. (1997). Nutritional and toxic factors in selected wild edible plants. Plant Foods Hum. Nutr..

[22] Kristanc L., Kreft S. (2016). European medicinal and edible plants associated with subacute and chronic toxicity part I: Plants with carcinogenic, teratogenic and endocrine-disrupting effects. Food Chem. Toxicol..

[23] Kristanc L., Kreft S. (2016). European medicinal and edible plants associated with subacute and chronic toxicity part II: Plants with hepato-, neuro-, nephro- and immunotoxic effects. Food Chem. Toxicol..

[24] Della A., Paraskeva-Hadjichambi D., Hadjichambis A.C. (2006). An ethnobotanical survey of wild edible plants of Paphos and Larnaca countryside of Cyprus. J. Ethnobiol. Ethnomed..

[25] Kubacey T., Haggag E., El-Toumy S., Ahmed A., El-Ashmawy I., Youns M. (2012). Biological activity and flavonoids from *Centaurea alexanderina* leaf extract. J. Pharm. Res..

[26] Pieroni A., Janiak V., Dürr C.M., Lüdeke S., Trachsel E., Heinrich M. (2002). In vitro antioxidant activity of non-cultivated vegetables of ethnic Albanians in southern Italy. Phyther. Res..

[27] Csupor D., Widowitz U., Blazsõ G., Laczkõ-Zöld E., Tatsimo J.S.N., Balogh Á., Boros K., Dankõ B., Bauer R., Hohmann J. (2013). Anti-inflammatory activities of eleven *Centaurea* species occurring in the Carpathian basin. Phyther. Res..

[28] Tekeli Y., Zengin G., Aktumsek A., Mehmet S., Torlak E. (2011). Antibacterial activities of extracts from twelve *Centaurea* species from Turkey. Arch. Biol. Sci..

[29] Lockowandt L., Pinela J., Roriz C.L., Pereira C., Abreu R.M.V., Calhelha R.C., Alves M.J., Barros L., Bredol M., Ferreira I.C.F.R. (2019). Chemical features and bioactivities of cornflower (*Centaurea cyanus* L.) capitula: The blue flowers and the unexplored non-edible part. Ind. Crop. Prod..

[30] Escher G.B., Santos J.S., Rosso N.D., Marques M.B., Azevedo L., do Carmo M.A.V., Daguer H., Molognoni L., Prado-Silva L.d., Sant’Ana A.S. (2018). Chemical study, antioxidant, anti-hypertensive, and cytotoxic/cytoprotective activities of *Centaurea cyanus* L. petals aqueous extract. Food Chem. Toxicol..

[31] Erol-Dayi Ö., Pekmez M., Bona M., Aras-Perk A., Arda N. (2011). Total phenolic contents, antioxidant activities cytotoxicity of three *Centaurea* species: *C. calcitrapa* subsp. *calcitrapa*, *C. ptosimopappa C. spicata*. Free Radic. Antioxid..

[32] Ostad S.N., Rajabi A., Khademi R., Farjadmand F., Eftekhari M., Hadjiakhoondi A., Khanavi M. (2016). Cytotoxic potential of *Centaurea bruguierana* ssp. *belangerana*: The MTT assay. Acta Med. Iran..

[33] Ifantis T.M., Solujić S., Pavlović-Muratspahić D., Skaltsa H. (2013). Secondary metabolites from the aerial parts of Centaurea pannonica (Heuff.) Simonk. from Serbia and their chemotaxonomic importance. Phytochemistry.

[34] Ćiricć A., Karioti A., Koukoulitsa C., Sokovic̈ M., Skaltsa H. (2012). Sesquiterpene lactones from Centaurea zuccariniana and their antimicrobial activity. Chem. Biodivers..

[35] Özcan K., Acet T., Çorbacı C. (2019). *Centaurea hypoleuca* DC: Phenolic content, antimicrobial, antioxidant and enzyme inhibitory activities. South Afr. J. Bot..

[36] Fernandes L., Pereira J.A., Saraiva J.A., Ramalhosa E., Casal S. (2019). Phytochemical characterization of *Borago officinalis* L. and *Centaurea cyanus* L. during flower development. Food Res. Int..

[37] Aktumsek A., Zengin G., Guler G.O., Cakmak Y.S., Duran A. (2013). Assessment of the antioxidant potential and fatty acid composition of four *Centaurea* L. taxa from Turkey. Food Chem..

[38] Erdogan T., Gonenc T., Cakilcioglu U., Kivcak B. (2014). Fatty acid composition of the aerial parts of some *Centaurea* species in Elazig, Turkey. Trop. J. Pharm. Res..

[39] Panagouleas C., Skaltsa H., Lazari D., Skaltsounis A.L., Sokovic M. (2003). Antifungal activity of secondary metabolites of *Centaurea raphanina* ssp. *mixta*, growing wild in Greece. Pharm. Biol..

[40] Petropoulos S.A., Fernandes Â., Dias M.I., Pereira C., Calhelha R., Gioia F.D., Tzortzakis N., Ivanov M., Sokovic M., Barros L. (2020). Wild and cultivated *Centaurea raphanina* subsp. *mixta*: A valuable source of bioactive compounds. Antioxidants.

[41] Negaresh K., Rahiminejad M.R. (2014). A contribution to the taxonomy of *Centaurea* sect. *Cynaroides* (Asteraceae, Cardueae-Centaureinae) in Iran. Phytotaxa.

[42] Hilpold A., Garcia-Jacas N., Vilatersana R., Susanna A. (2014). Taxonomical and nomenclatural notes on *Centaurea*: A proposal of classification, a description of new sections and subsections, and a species list of the redefined section *Centaurea*. Collect. Bot..

[43] Trichopoulou A., Vasilopoulou E., Hollman P., Chamalides C., Foufa E., Kaloudis T., Kromhout D., Miskaki P., Petrochilou I., Poulima E. (2000). Nutritional composition and flavonoid content of edible wild greens and green pies: A potential rich source of antioxidant nutrients in the Mediterranean diet. Food Chem..

[44] Conforti F., Sosa S., Marrelli M., Menichini F., Statti G.A., Uzunov D., Tubaro A., Menichini F. (2009). The protective ability of Mediterranean dietary plants against the oxidative damage: The role of radical oxygen species in inflammation and the polyphenol, flavonoid and sterol contents. Food Chem..

[45] Mikropoulou E.V., Vougogiannopoulou K., Kalpoutzakis E., Sklirou A.D., Skaperda Z., Houriet J.l., Wolfender J.L., Trougakos I.P., Kouretas D., Halabalaki M. (2018). Phytochemical composition of the decoctions of Greek edible greens (chórta) and evaluation of antioxidant and cytotoxic properties. Molecules.

[46] Fernández-Marín B., Milla R., Martín-Robles N., Arc E., Kranner I., Becerril J.M., García-Plazaola J.I. (2014). Side-effects of domestication: Cultivated legume seeds contain similar tocopherols and fatty acids but less carotenoids than their wild counterparts. Bmc Plant Biol..

[47] Nemzer B., Al-Taher F., Abshiru N. (2020). Phytochemical composition and nutritional value of different plant parts in two cultivated and wild purslane (*Portulaca oleracea* L.) genotypes. Food Chem..

[48] Karkanis A.C., Fernandes A., Vaz J., Petropoulos S., Georgiou E., Ciric A., Sokovic M., Oludemi T., Barros L., Ferreira I. (2019). Chemical composition and bioactive properties of *Sanguisorba minor* Scop. under Mediterranean growing conditions. Food Funct..

[49] Petropoulos S., Fernandes Â., Vasileios A., Ntatsi G., Barros L., Ferreira I.I.C.F.R., Antoniadis V., Ntatsi G., Barros L., Ferreira I.I.C.F.R. (2018). Chemical composition and antioxidant activity of *Cichorium spinosum* L. leaves in relation to developmental stage. Food Chem..

[50] Cros V., Martínez-Sánchez J.J., Franco J.A. (2007). Good yields of common purslane with a high fatty acid content can be obtained in a peat-based floating system. Horttechnology.

[51] Petropoulos S.A., Karkanis A., Martins N., Ferreira I.C.F.R. (2016). Phytochemical composition and bioactive compounds of common purslane (*Portulaca oleracea* L.) as affected by crop management practices. Trends Food Sci. Technol..

[52] Loconsole D., Cristiano G., De Lucia B. (2019). Glassworts: From wild salt marsh species to sustainable edible crops. Agriculture.

[53] Petropoulos S.A., Pereira C., Tzortzakis N., Barros L., Ferreira I.C.F.R. (2018). Nutritional value and bioactive compounds characterization of plant parts from *Cynara cardunculus* L. (Asteraceae) cultivated in central Greece. Front. Plant Sci..

[54] Montesano F.F., Gattullo C.E., Parente A., Terzano R., Renna M. (2018). Cultivation of potted sea fennel, an emerging mediterranean halophyte, using a renewable seaweed-based material as a peat substitute. Agriculture.

[55] Bonasia A., Lazzizera C., Elia A., Conversa G. (2017). Nutritional, biophysical and physiological characteristics of wild rocket genotypes as affected by soilless cultivation system, salinity level of nutrient solution and growing period. Front. Plant Sci..

[56] Petropoulos S.A., Daferera D., Polissiou M.G., Passam H.C. (2009). The effect of salinity on the growth, yield and essential oils of turnip-rooted and leaf parsley cultivated within the Mediterranean region. J. Sci. Food Agric..

[57] Rouphael Y., Petropoulos S.A., Cardarelli M., Colla G. (2018). Salinity as eustressor for enhancing quality of vegetables. Sci. Hortic. (Amst. )..

[58] Radić S., Radić-Stojković M., Pevalek-Kozlina B. (2006). Influence of NaCl and mannitol on peroxidase activity and lipid peroxidation in *Centaurea ragusina* L. roots and shoots. J. Plant Physiol..

[59] Radić S., Prolić M., Pavlica M., Pevalek-Kozlina B. (2005). Cytogenetic effects of osmotic stress on the root meristem cells of *Centaurea ragusina* L.. Env. Exp. Bot..

[60] Nosratti I., Soltanabadi S., Honarmand S.J., Chauhan B.S. (2017). Environmental factors affect seed germination and seedling emergence of invasive *Centaurea balsamita*. Crop. Pasture Sci..

[61] Lidón A., Boscaiu M., Collado F., Vicente O. (2009). Soil requirements of three salt tolerant, endemic species from south-east Spain. Not. Bot. Horti Agrobot. Cluj-Napoca.

[62] Abusaief H., Husien D., Naby A. (2013). Al Salinity tolerance of the flora halophytes to coastal habitat of Jarjr-oma in Libya. Nat. Sci..

[63] Yimathldimathztugay E., Sekmen A.H., Turkan I., Kucukoduk M. (2011). Elucidation of physiological and biochemical mechanisms of an endemic halophyte *Centaurea tuzgoluensis* under salt stress. Plant Physiol. Biochem..

[64] Rouphael Y., Kyriacou M.C. (2018). Enhancing quality of fresh vegetables through salinity eustress and biofortification applications facilitated by soilless cultivation. Front. Plant Sci..

[65] Fallovo C., Rouphael Y., Rea E., Battistelli A., Colla G. (2009). Nutrient solution concentration and growing season affect yield and quality of *Lactuca sativa* L. var. acephala in floating raft culture. J. Sci. Food Agric..

[66] Di Gioia F., Rosskopf E.N., Leonardi C., Giuffrida F. (2018). Effects of application timing of saline irrigation water on broccoli production and quality. Agric. Water Manag..

[67] Colla G., Rouphael Y., Cardarelli M., Svecova E., Rea E., Lucini L. (2013). Effects of saline stress on mineral composition, phenolic acids and flavonoids in leaves of artichoke and cardoon genotypes grown in floating system. J. Sci. Food Agric..

[68] Tarchoune I., Sgherri C., Baâtour O., Izzo R., Lachaâl M., Navari-Izzo F., Ouerghi Z. (2013). Effects of oxidative stress caused by NaCl or Na2SO4 excess on lipoic acid and tocopherols in Genovese and Fine basil (*Ocimum basilicum*). Ann. Appl. Biol..

[69] Klados E., Tzortzakis N. (2014). Effects of substrate and salinity in hydroponically grown *Cichorium spinosum*. J. Soil Sci. Plant Nutr..

[70] Chennupati P., Seguin P., Liu W. (2011). Effects of high temperature stress at different development stages on soybean isoflavone and tocopherol concentrations. J. Agric. Food Chem..

[71] Wojciechowska R., Dugosz-Grochowska O., Koton A., Zupnik M. (2015). Effects of LED supplemental lighting on yield and some quality parameters of lamb’s lettuce grown in two winter cycles. Sci. Hortic..

[72] Rosa M., Prado C., Podazza G., Interdonato R., González J.A., Hilal M., Prado F.E. (2009). Soluble sugars-metabolism, sensing and abiotic stress a complex network in the life of plants. Plant Signal. Behav..

[73] Poli F., Sacchetti G., Tosi B., Fogagnolo M., Chillemi G., Lazzarin R., Bruni A. (2002). Variation in the content of the main guaianolides and sugars in *Cichorium intybus* var. “Rosso di Chioggia” selections during cultivation. Food Chem..

[74] Sokovic M., Ciric A., Glamoclija J., Skaltsa H. (2017). Biological activities of sesquiterpene lactones isolated from the genus *Centaurea* L. (Asteraceae). Curr. Pharm. Des..

[75] Shakeri A., Masullo M., Bottone A., Asili J., Emami S.A., Piacente S., Iranshahi M. (2019). Sesquiterpene lactones from *Centaurea rhizantha* C.A. Meyer. Nat. Prod. Res..

[76] Blom-Zandstra M., Lampe J.E.M., Ammerlaan F.H.M. (1988). C and N utilization of two lettuce genotypes during growth under non-varying light conditions and after changing the light intensity. Physiol. Plant..

[77] Zhang Y., Lin X., Zhang Y., Zheng S.J., Du S. (2005). Effects of nitrogen levels and nitrate/ammonium ratios on oxalate concentrations of different forms in edible parts of spinach. J. Plant Nutr..

[78] Fujii N., Watanabe M., Watanabe Y., Shimada N. (1993). Rate of oxalate biosynthesis from glycolate and ascorbic acid in spinach leaves. Soil Sci. Plant Nutr..

[79] Tekeli Y., Sezgin M., Aktumsek A., Ozmen Guler G., Aydin Sanda M. (2010). Fatty acid composition of six *Centaurea* species growing in Konya, Turkey. Nat. Prod. Res..

[80] Aktumsek A., Zengin G., Guler G.O., Cakmak Y.S., Duran A. (2011). Screening for in vitro antioxidant properties and fatty acid profiles of five *Centaurea* L. species from Turkey flora. Food Chem. Toxicol..

[81] Cao J., Li H., Xia X., Zou X.-G., Li J., Zhu X.-M., Deng Z.-Y. (2015). Effect of fatty acid and tocopherol on oxidative stability of vegetable oils with limited air. Int. J. Food Prop..

[82] Maeda H., Sage T.L., Isaac G., Welti R., Dellapenna D. (2008). Tocopherols modulate extraplastidic polyunsaturated fatty acid metabolism in Arabidopsis at low temperature. Plant Cell.

[83] Guil J.L., Torija M.E., Giménez J.J., Rodriguez I. (1996). Identification of fatty acids in edible wild plants by gas chromatography. J. Chromatogr. A.

[84] Dalar A., Uzun Y., Mukemre M., Turker M., Konczak I. (2015). *Centaurea karduchorum* Boiss. from Eastern Anatolia: Phenolic composition, antioxidant and enzyme inhibitory activities. J. Herb. Med..

[85] Petropoulos S.A., Fernandes Â., Tzortzakis N., Sokovic M., Ciric A., Barros L., Ferreira I.C.F.R. (2019). Bioactive compounds content and antimicrobial activities of wild edible Asteraceae species of the Mediterranean flora under commercial cultivation conditions. Food Res. Int..

[86] Alam M.A., Juraimi A.S., Rafii M.Y., Hamid A.A., Aslani F., Alam M.Z. (2015). Effects of salinity and salinity-induced augmented bioactive compounds in purslane (*Portulaca oleracea* L.) for possible economical use. Food Chem..

[87] Sarker U., Oba S. (2019). Salinity stress enhances color parameters, bioactive leaf pigments, vitamins, polyphenols, flavonoids and antioxidant activity in selected *Amaranthus* leafy vegetables. J. Sci. Food Agric..

[88] Sarker U., Islam M.T., Oba S. (2018). Salinity stress accelerates nutrients, dietary fiber, minerals, phytochemicals and antioxidant activity in *Amaranthus tricolor* leaves. PLoS ONE.

[89] Neffati M., Sriti J., Hamdaoui G., Kchouk M.E., Marzouk B. (2011). Salinity impact on fruit yield, essential oil composition and antioxidant activities of *Coriandrum sativum* fruit extracts. Food Chem..

[90] Boulaaba M., Abdelly C., Abdelly C., Öztürk M., Ashraf M., Grignon C. (2008). Effect of salinity on growth, leaf-phenolic content and antioxidant scavenging activity in Cynara cardunculus L.. Biosaline Agriculture and Salinity Tolerance in Plants.

[91] Azzi A., Stocker A. (2000). Vitamin E: Non-antioxidant roles. Prog. Lipid Res..

[92] Freyer M.J. (1992). The antioxidant effects of thylakoid Vitamin E (α-tocopherol). Plant. Cell Env...

[93] Keles Y., Oncel I. (2002). Response of antioxidative defence system to temperature and water stress combinations in wheat seedlings. Plant Sci..

[94] Morales P., Carvalho A.M., Sánchez-Mata M.C., Cámara M., Molina M., Ferreira I.C.F.R. (2012). Tocopherol composition and antioxidant activity of Spanish wild vegetables. Genet. Resour. Crop. Evol..

[95] Kollia E., Markaki P., Zoumpoulakis P., Proestos C. (2016). Antioxidant activity of *Cynara scolymus* L. and *Cynara cardunculus* L. extracts obtained by different extraction techniques. Nat. Prod. Res..

[96] Petropoulos S.A., Pereira C., Barros L., Ferreira I.C.F.R. (2017). Leaf parts from Greek artichoke genotypes as a good source of bioactive compounds and antioxidants. Food Funct..

[97] Karamenderes C., Konyalioglu S., Khan S., Khan I.A. (2007). Total phenolic contents, free radical scavenging activities and inhibitory effects on the activation of NF-kappa B of eight *Centaurea* L. species. Phyther. Res..

[98] Cappadone C., Mandrone M., Chiocchio I., Sanna C., Malucelli E., Bassi V., Picone G., Poli F. (2019). Antitumor potential and phytochemical profile of plants from sardinia (Italy), a hotspot for biodiversity in the mediterranean basin. Plants.

[99] Koukoulitsa C., Geromichalos G.D., Skaltsa H. (2005). VolSurf analysis of pharmacokinetic properties for several antifungal sesquiterpene lactones isolated from Greek *Centaurea* sp. J. Comput. Aided. Mol. Des..

[100] Lahneche A.M., Boucheham R., Ozen T., Altun M., Boubekri N., Demirtas I., Bicha S., Bentamene A.L.I., Benayache F., Benayache S. (2019). In vitro antioxidant, dna-damaged protection and antiproliferative activities of ethyl acetate and n-butanol extracts of *Centaurea sphaerocephala* L.. . Acad. Bras. Cienc..

[101] Karioti A., Skaltsa H., Lazari D., Sokovic M., Garcia B., Harvala C. (2002). Secondary metabolites from *Centaurea deusta* with antimicrobial activity. Z. Fur Nat. Sect. C J. Biosci..

[102] D’Antuono I., Garbetta A., Linsalata V., Minervini F., Cardinali A. (2015). Polyphenols from artichoke heads (*Cynara cardunculus* (L.) subsp. *scolymus* Hayek): In vitro bio-accessibility, intestinal uptake and bioavailability. Food Funct..

[103] Pereira C., Calhelha R.C., Barros L., Queiroz M.J.R.P., Ferreira I.C.F.R. (2014). Synergisms in antioxidant and anti-hepatocellular carcinoma activities of artichoke, milk thistle and borututu syrups. Ind. Crop. Prod..

[104] Horwitz W., Latimer G., AOAC (2016). Official methods of analysis of AOAC International. Official Methods of Analysis of AOAC International.

[105] Guimarães R., Barros L., Dueñas M., Calhelha R.C., Carvalho A.M., Santos-Buelga C., Queiroz M.J.R.P., Ferreira I.C.F.R. (2013). Nutrients, phytochemicals and bioactivity of wild Roman chamomile: A comparison between the herb and its preparations. Food Chem..

[106] Pereira C., Barros L., Carvalho A.M., Ferreira I.C.F.R. (2013). Use of UFLC-PDA for the analysis of organic acids in thirty-five species of food and medicinal plants. Food Anal. Methods.

[107] Bessada S.M.F., Barreira J.C.M., Barros L., Ferreira I.C.F.R., Oliveira M.B.P.P. (2016). Phenolic profile and antioxidant activity of *Coleostephus myconis* (L.) Rchb.f.: An underexploited and highly disseminated species. Ind. Crop. Prod..

[108] Abreu R.M.V., Ferreira I.C.F.R., Calhelha R.C., Lima R.T., Vasconcelos M.H., Adega F., Chaves R., Queiroz M.J.R.P. (2011). Anti-hepatocellular carcinoma activity using human HepG2 cells and hepatotoxicity of 6-substituted methyl 3-aminothieno[3,2-b]pyridine-2- carboxylate derivatives: In vitro evaluation, cell cycle analysis and QSAR studies. Eur. J. Med. Chem..

[109] Sokovic M., Glamočlija J., Marin P.D., Brkić D., Van Griensven L.J.L.D. (2010). Antibacterial effects of the essential oils of commonly consumed medicinal herbs using an in vitro model. Molecules.

